# Treatment of Canine Leishmaniasis with Meglumine Antimoniate: A Clinical Study of Tolerability and Efficacy

**DOI:** 10.3390/ani14152244

**Published:** 2024-08-01

**Authors:** Serena Digiaro, Alessandra Recchia, Antonella Colella, Sara Cucciniello, Beatrice Greco, Dora Buonfrate, Paola Paradies

**Affiliations:** 1 Department DiMePre-J, Veterinary Section, University of Bari “Aldo Moro”, Strada Provinciale Casamassima km3, Valenzano, 70010 Bari, Italy; serena.digiaro@uniba.it (S.D.); alessandra.recchia1@uniba.it (A.R.); antonella.colella@uniba.it (A.C.); s-cucciniello@libero.it (S.C.); beatrice.greco@uniba.it (B.G.); 2 Department of Infectious, Tropical Diseases and Microbiology, IRCCS Sacro Cuore Don Calabria Hospital, Negrar, 37024 Verona, Italy; dora.buonfrate@sacrocuore.it

**Keywords:** canine leishmaniasis, meglumine antimoniate, adverse effects, efficacy

## Abstract

**Simple Summary:**

This study evaluates the potential adverse effects associated with meglumine antimoniate (aNm) in the treatment of canine leishmaniasis through both a retrospective analysis and a prospective study. The retrospective study comprised records of 87 dogs treated with aNm. Adverse effects during treatment emerged in about one third of dogs, including local reactions at the injection site, injection site pain-related systemic reaction, systemic disease due to renal function worsening, acute pancreatitis, gastrointestinal self-limiting signs or severe idiosyncratic skin reactions. Half of these animals required treatment suspension. The prospective study included 16 dogs (LeishVet stages II and III) treated with the same aNm; of these, 2 dogs were excluded for severe reactions at the injection site and 4 dogs reported mild and transient adverse events. In the prospective study, treatment efficacy was also evaluated at 1-year follow-up. No animals showed clinical laboratory relapse during the study and interestingly, PCR turned negative between D0 and D60 in 78.5% of animals. Adverse severe events associated with aNm are possible, but the high rate of parasitic clearance supports its use as first line treatment, at least in dogs with normal renal function.

**Abstract:**

Antimoniate therapy, in association with allopurinol, is one of the first-line treatments of canine leishmaniasis (CanL). This study evaluates the potential adverse effects associated with aNm in the treatment of CanL through both a retrospective analysis and a long-term prospective study also aimed to investigate its efficacy. The retrospective study reviewed records of 87 dogs with CanL with at least one follow-up available during or at the end of therapy with aNm (Glucantime^®^) at a dose of 50 mg/kg administered subcutaneously twice a day in association with allopurinol. In total, 29.8% of dogs showed adverse effects during treatment as local reactions at the injection site (n = 6), severe systemic reaction to pain (originating from the inoculation site) with depression and anorexia (n = 4), systemic disease due to renal function worsening (n = 4), acute pancreatitis (n = 1), diarrhea (n = 5), vomiting (n = 3) and severe idiosyncratic skin reactions (n = 3). Of these dogs, 13 (14.9%) required treatment suspension. The prospective study included 16 dogs, selected among the LeishVet stages II and III CKD IRIS stage 1 (International Renal Interest Society staging of canine Chronic Kidney Disease) and treated with the same aNm plus allopurinol protocol as in the retrospective study and observed for 360 days; 2 dogs were excluded for severe reactions at the injection site. Mild and transient adverse events were reported in the other 4 dogs. The criteria used to evaluate the efficacy of treatment with aNm were as follows: a reduction in the clinical score and improvement and/or normalization of laboratory parameters, negativization of PCR on the bone marrow samples and disease-free interval time. The proportion of reduction in the clinical score reached 91.9% at D180. No animals showed clinical laboratory relapse during the whole study duration and interestingly, the PCR results showed complete negativity between D0 and D60 in 78.5% of animals. Veterinarians must be vigilant regarding the potentially serious adverse effects associated with aNm and promptly stop drug administration if unexpected clinical manifestations occur. On the other hand, they should not discard its use for CanL treatment since it is confirmed that aNm in association with allopurinol is highly effective in controlling CanL.

## 1. Introduction

Canine leishmaniasis (CanL) is a protozoal disease caused by *Leishmania infantum*. The life cycle of *L. infantum* involves definitive mammal hosts and vector insects, including the sand fly [1]. CanL is endemic in the Mediterranean basin, South America, and central and southwestern Asia [1]. *L. infantum* commonly causes chronic infection with subclinical or clinical course, depending on parasite strain, host genetics and immune status [2]. Based on this, some dogs will be able to control the infection for many years without clinical signs. On the other hand, some infected dogs may present acute evolution and severe disease, or a progressive disease that could be fatal if proper management and supportive therapy are not promptly provided [2].

Several pharmacological treatment protocols have been proposed for CanL. The purpose of CanL treatment is to control clinical signs and hematobiochemical alterations, improve the dog’s cellular immunity, reduce the parasitic load, prevent relapses, and decrease the transmission rate to the vector [3]. It is common opinion that treatment can lead to parasitological clearance, though this is not frequent [1,4,5]. Consequently, clinical remission may only be temporary [1,6] and clinical relapse can occur, although reinfection is always possible in endemic areas. First-choice treatments used for CanL are the combinations of N-methylglucamine antimoniate (aNm) plus allopurinol and miltefosine (MIL) plus allopurinol [1]. Although aNm is a much older and effective treatment for CanL, upon introduction in the veterinary market in the last 15 years, MIL has increasingly been used by veterinarians with some undeniable advantages, such as the once-a-day oral administration and the mild adverse effects, which are mostly self-limiting and result in dose-dependent gastrointestinal reactions [7]. In this study, we explored whether there was enough evidence to suggest treatment with MIL instead of treatment with aNm in all dogs affected by CanL. Several authors have compared the efficacy of aNm and allopurinol treatment with MIL and allopurinol, finding similar clinical efficacy, although treatment response is faster [8,9] and clinical relapses are less frequent [9,10,11] when the aNm protocol is used. Other studies have suggested that aNm compared to MIL may be more effective in controlling inflammation and oxidation in CanL [12,13,14,15], while few comparing data are available on the parasitological clearance after treatment.

On the other hand, the possibility of clinical adverse events associated to aNm treatment is known [1,16], but mostly based on fragmentary descriptions rather than on large-scale studies focusing on this aspect [6,9,17]. Furthermore, based on the current literature, the impact of aNm on renal function is controversial, with some authors concerned about potential impairment [8,17,18,19,20], and others suggesting the absence of renal involvement [6,9,12,21].

The purpose of this work was to report the potential clinical adverse events associated with the administration of aNm as well as the efficacy of the treatment with aNm plus allopurinol.

## 2. Materials and Methods

The aNm tolerability was evaluated with a retrospective and a prospective study, while the efficacy was estimated with the prospective study only. Procedures to confirm diagnosis and treatment protocol were the same both in the retrospective and prospective study (see below).

### 2.1. Retrospective Study

Clinical records of patients diagnosed with CanL, treated with aNm and allopurinol (standard protocol) and with at least one follow-up available during and/or immediately after aNm discontinuation were reviewed.

Dogs were included regardless of the LeishVet clinical stage. Dogs presented for consultation due to the appearance of new clinical signs during standard treatment set by a practitioner were also included. Exclusion criteria were as follows: (i) treatment with different aNm protocols; (ii) treatment with further drugs for concomitant diseases; (iii) severe concomitant diseases.

Available clinical records during the aNm 30-day treatment and/or the first post-aNm treatment record were reviewed to identify described or reported potential adverse events. Available collateral exams (i.e., laboratory and imaging examinations) were also reviewed to better understand the clinical picture in dogs presenting adverse events.

### 2.2. Prospective Study

Dogs with a CanL diagnosis of stage II or III (IRIS 1) according to the LeishVet classification [22] were included in the study, irrespective of breed, sex or age.

Exclusion criteria were as follows: (i) treatment with effective drugs against CanL in the previous 3 months; (ii) treatment with long-acting corticosteroids and other immunomodulatory drugs within 1 month before inclusion; (iii) concurrent disorders and potentially lethal diseases; (iv) pregnancy or lactation. Dogs were housed, fed regularly, and received treatment by owners. Dogs were tested for other vector-borne diseases (i.e., *Ehrlichia* spp.) and only negative dogs were included. Dogs were treated with the aNm plus allopurinol standard protocol.

Dogs whose pharmacological side effects required treatment interruption during follow-up evaluation were excluded. All possible side effects reported by owners were documented during the month of aNm therapy.

Dogs were observed for 360 days. Clinical scores and body weights were recorded on individual files at day 0, 30, 60, 90, 180, and 360 (D0–D360). Clinical scores were obtained by assessing the presence and severity of 26 clinical signs according to Mirò et al. [23]. Blood samples were collected at each follow-up to record the complete blood count (CBC), biochemical data (e.g., urea, creatinine, total plasma proteins, albumin concentration, globulin concentration and fractions, albumin/globulin ratio, protein electrophoresis, alanine-aminotransferase (ALT), alkaline phosphatase (ALP), plasma bilirubin), and *Leishmania* spp. IFAT. At D0 and D60, fine needle aspiration on popliteal lymph nodes was performed to microscopically identify *Leishmania* spp. amastigotes [24], and bone marrow samples were collected to perform qPCR analysis from sternum bones. Urine samples were collected under ultrasound-guide at D0, D30 and D60 to perform a complete urine analysis and to evaluate the urine protein/creatinine ratio (UPC). The study was approved by the ethics committee of the Department of Emergency and Organ Transplantation, Uniba (Prot. DETO 226/2018).

Treatment safety was assessed by the incidence of adverse events observed daily by the owner. Evaluation of urea, creatinine, and liver enzymes as well as the UPC ratio before and after treatment were also considered to assess treatment safety.

Clinical score reduction, improvement and/or normalization of laboratory parameters, serological data, PCR negativization on bone marrow, and disease-free interval time were used as criteria to assess treatment efficacy.

These parameters were evaluated during each follow-up in order to identify dogs under relapse. Numbers and times of relapses were assessed at the end of the study. Relapse was defined by the reappearance of clinical signs and/or clinical–pathological alterations indicative of leishmaniasis [3,22].

### 2.3. Diagnostic Procedures

In both retrospective and prospective studies, diagnosis was confirmed by cytological positivity in lymph node aspirates stained with May–Grüwndal–Giemsa [24] and observed under a microscope by an experienced operator and/or by IFAT (cut off dilution 1:80) in patients with a compatible, strong clinical suspicion. An in-house IFAT assay was performed using *L. infantum* promastigotes as the antigen, and following a previously recommended protocol [25].

Bone marrow fine needle aspirate material was examined a posteriori by q-PCR assay following the methodology used by the Italian National Reference Center for Leishmaniasis (C.Re.Na.L., Palermo, Italy). In brief, DNA from bone marrow was subjected to two consecutive PCR amplifications using the kinetoplast-specific primers R221 and R332 in the first run, and the *Leishmania*-specific primers R223 and R333 in the second run [26].

### 2.4. Treatment

Dogs were treated with aNm (Glucantime^®^, Boehringer, Milan, Italy) at a dose of 50 mg/kg/twice a day (BID) subcutaneously (SC) for 30 days and allopurinol (Zyloric^®^, Teofarma, Pavia, Italy) at a dosage of 10 mg/kg/BID per os (OS) for 6 months (standard protocol) in both retrospective and prospective studies. As a matter of practice, at the time of diagnosis, the owners were instructed to use the following injection pattern: left side of midline in the morning; right side in the evening moving cranio-caudally from the neck to the thorax each day to avoid proximity between injections. In the retrospective study, only dogs under the standard aNm protocol were included to avoid variability due to dosage.

## 3. Results

### 3.1. Retrospective Study

**Figure 1:**
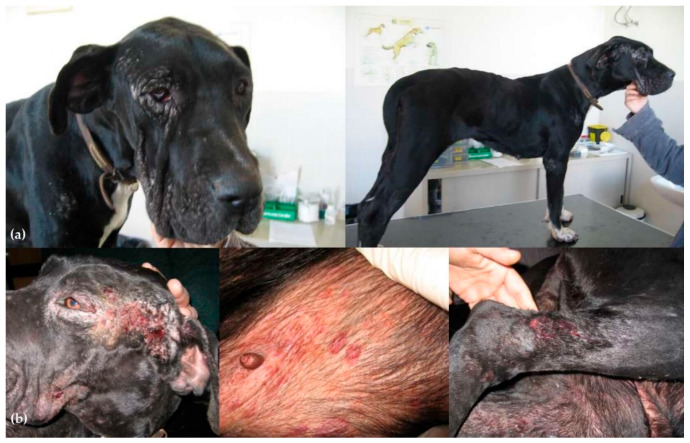
Meglumine antimoniate (aNm) cutaneous idiosyncratic reaction on a 3-year-old male Great Dane. (**a**) Mild clinical signs at the time of diagnosis before aNm treatment started; (**b**) acute onset of facial and ventral erythema and erosion, which appeared 3 days after aNm treatment started and rapidly progressed until aNm was stopped at day 7. Pictures (**b**) refer to the day of consultation (i.e., day 7).

**Figure 2:**
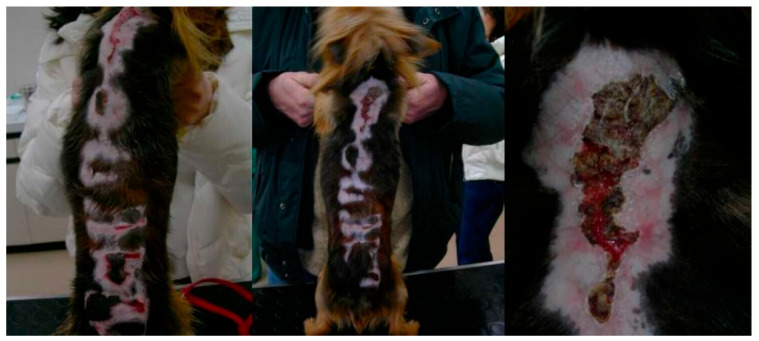
A 5-year-old female Yorkshire. Progressive serpiginous cutaneous lesion on the back with a burn-like appearance that manifested during aNm treatment.

**Figure 3:**
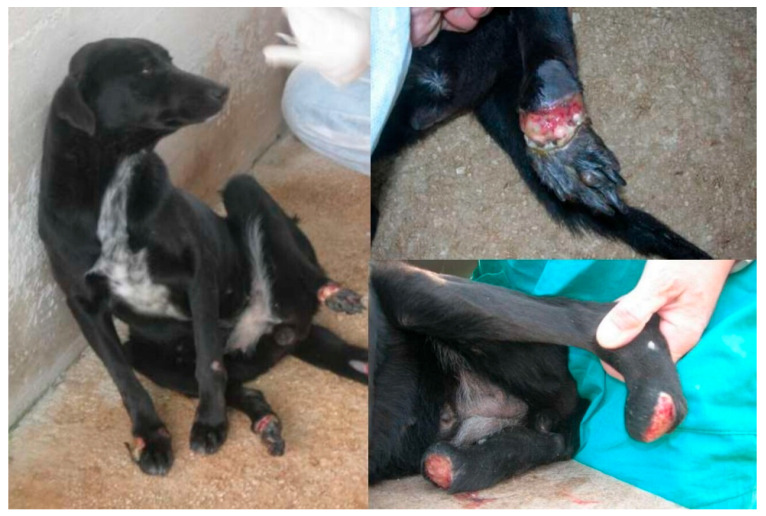
A 6-year-old female mongrel. Deep linear skin ulcer on all limbs that appeared after aNm treatment started. Evolution until complete detachment of the feet despite treatment suspension.

The records of 87 dogs were reviewed. Data on signalment, presenting complaints, and clinical signs at the time of diagnosis are reported in Table 1 and Table 2. Diagnosis was achieved through lymph node cytology and IFAT in 80/87 dogs and with just IFAT in 7 dogs. Clinical signs and pathological conditions occurring during treatment and associated with aNm adverse events are reported in Table 3. In brief, 26 (29.8%) animals showed clinical evidence of adverse events of various types and severity. Treatment suspension was necessary in 13 animals (14.9%) (Table 3). In particular, six (6.9%) dogs showed a local reaction at the inoculation site (swelling/granuloma/abscess), which required interruption of treatment in one case; four (4.6%) dogs showed a severe systemic reaction to pain (originating from the inoculation site) and characterized by lethargy, anorexia and reluctance to move (with normal blood test results and with return to normality within a few days after treatment discontinuation); five patients developed clinical signs associated with renal failure (4.6%) and acute pancreatitis (0.87%); five (5.7%) patients manifested diarrhea and three (3.4%) vomiting during treatment. Severe cutaneous reactions were reported in three other patients that needed immediate aNm discontinuation and were reported as idiosyncratic reactions. In particular, a 3-year-old male Great Dane presented with acute onset of facial and ventral erythema, itching, and erosions (Figure 1), which appeared 3 days after treatment and worsened (consultation was carried out after 7 days from treatment onset); a 5-year-old female Yorkshire presented to consultation for a progressive serpiginous cutaneous lesion on their back with a burn-like appearance (Figure 2) that manifested during treatment; a 6-year-old medium-sized female mongrel initially presented with a deep linear skin ulcer on one limb, which, in a few days, extended to all four limbs and led to complete detachment of the foot despite treatment suspension (Figure 3).

### 3.2. Prospective Study

**Figure 4:**
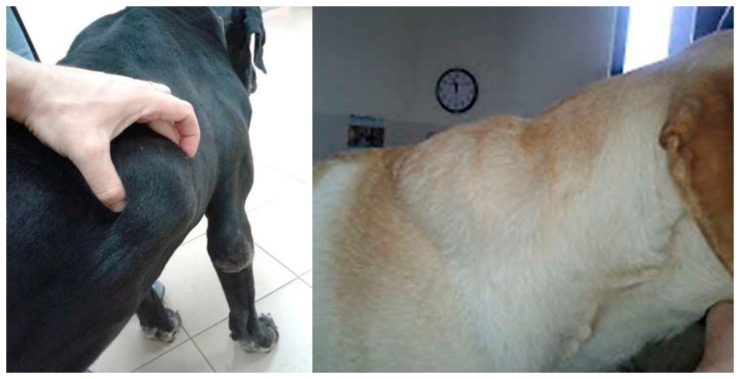
Severe local reaction (granuloma and abscess) after aNm treatment started in two dogs (C5 and C12) that led to their exclusion from the prospective study before D30.

Sixteen dogs met the inclusion criteria and were enrolled. The dogs’ data, clinical score, laboratory parameters, and LeishVet classification at the time of inclusion are reported in Table 4. Nine dogs were classified as stage II, and seven as stage III, IRIS 1. Two dogs were excluded from the study before D30 due to a severe local reaction (Figure 4); thus, they were treated with an alternative drug (i.e., MIL) (dog 5 and 12). The remaining 14 dogs presented the following adverse events during treatment: a mild and transient subcutaneous reaction appearing in the first week of treatment and self-limiting in 2 dogs (dog 13 and 16), episodic recurrent diarrhea throughout the treatment duration (dog 6), laboratory alterations indicative of liver impairment (ALT 478 IU/L 20–75, AST 301 IU/L 18–50, bilirubin 1.25 mg/dL 0.10–0.30) in the absence of clinical signs at D30 and self-limiting (dog 3).

**Figure 5:**
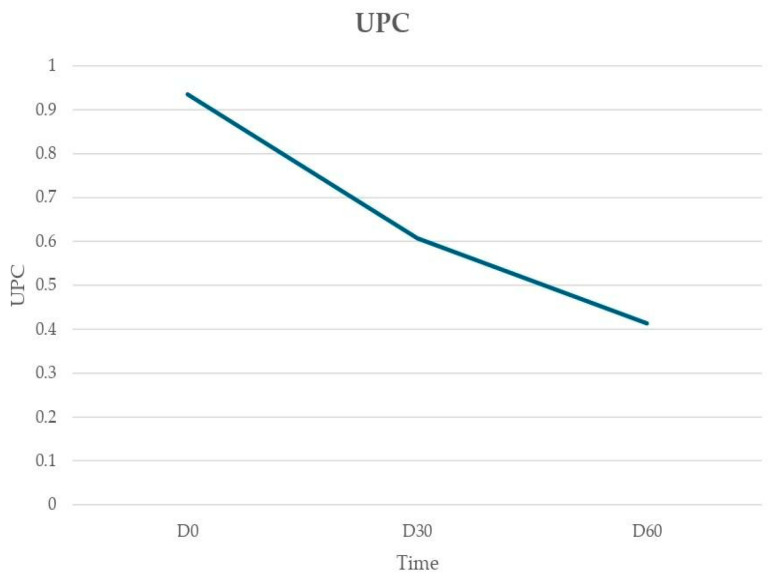
Urine protein/creatinine ratio (UPC) trend assessed at D0, D30 and D60.

Table 5 displays the parameters of renal function in dogs under aNm treatment, with urea, creatinine, and UPC values at D0, D30, and D60. No animal showed alterations in urea or creatinine parameters during or after therapy; UPC did not worsen in any of the dogs. In all the 6 dogs with proteinuria (UPC > 1) at D0 (LeishVet stage III CKD IRIS 1), a reduction in UPC was registered. The UPC trend is reported in Figure 5.

**Figure 6:**
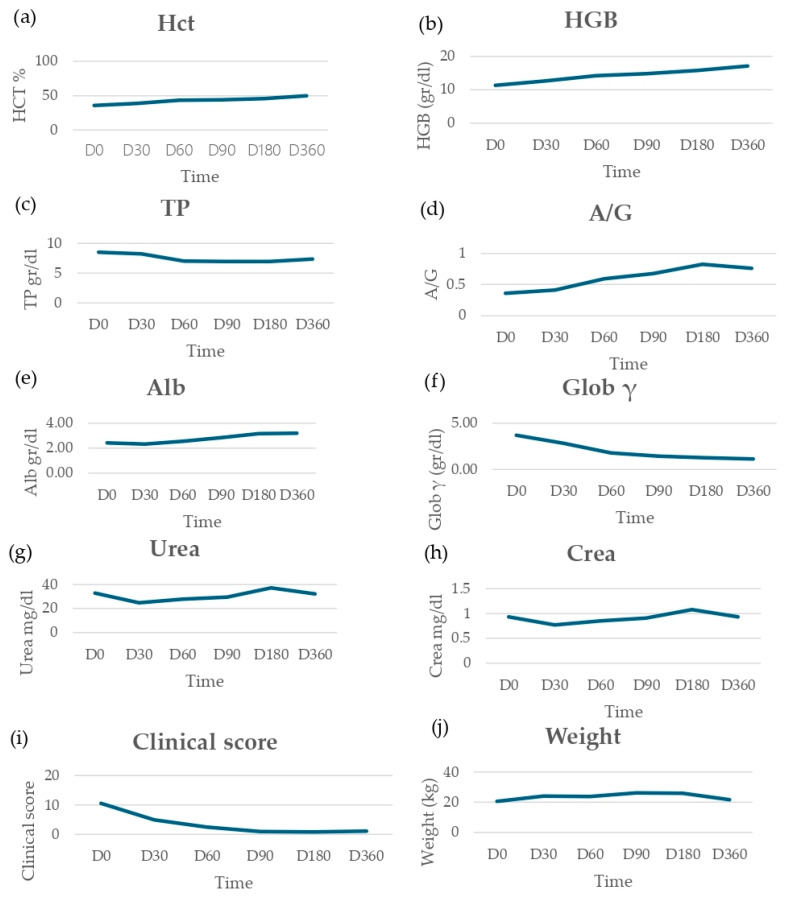
Selected clinical and hematobiochemical parameters trend assessed throughout the study, from D0 to D360. (**a**) Hematocrit (Hct) trend; (**b**) hemoglobin (HGB) trend; (**c**) total protein (TP) trend; (**d**) albumin/globulin ratio (A/G) trend; (**e**) albumin (Alb) trend; (**f**) gamma globulin (Glob γ) trend; (**g**) urea trend; (**h**) creatinine (Crea) trend; (**i**) clinical score trend; (**j**) weight trend.

The clinical score decreased from 10.64 (average value) to 0.86 from D0 to D180, while the average weight of the 14 dogs included in the study increased from 20.05 to 26 kg. The clinical score showed a 53.7% reduction from D0 to D30, reaching 91.9% at D180. The trend of clinical and laboratory parameters through the six time points is reported in Figure 6. None of the dogs showed clinical/laboratory relapse until the end of the study. In all dogs, IFAT titers decreased or remained stable during the study, but none of the patients showed serological negative results. Lymph node cytology, which was positive in all dogs at D0, turned negative in all dogs at D60. PCR results on bone marrow showed complete parasitological clearance between D0 and D60 in 11/14 animals (Table 6).

## 4. Discussion

Potential adverse events associated with aNm treatment were registered in nearly one-third (29.8%) of dogs in the retrospective study. This percentage may have been overestimated, since it includes seven dogs that manifested self-limiting vomiting and diarrhea where dietary indiscretions cannot be completely excluded due to the retrospective nature of the study. Furthermore, the percentage of adverse events decreases to 14.9%, if severe adverse events requiring treatment discontinuation are considered.

The most frequently reported clinical events include apathy, anorexia, vomiting, diarrhea, and pain at the inoculation site [6,9,19,27]. Increased liver transaminases (for transient hepatotoxicity in the absence of clinical signs), and decreased iron levels have also been described [1,16]. It has also been hypothesized that aNm may cause cardiotoxicity (arrhythmias, QT prolongation, and sudden death) in human patients with various forms of leishmaniasis treated with antimonials [28,29]. However, Xenoulis et al. [30] compared serum concentrations of troponin, a marker of heart disease, in dogs with leishmaniasis treated either with aNm or with another drug, showing that no dogs developed signs of cardiotoxicity.

Local reaction at the inoculation site was the most common side effect in the retrospective study, but treatment discontinuation was needed just in one dog. In the prospective study, two dogs developed a severe local reaction resulting in their exclusion from the study, with one requiring surgical curettage. Local reactions were described as warm and painful edematous areas and granulomas localized at the injection sites. As mentioned, these reactions occurred although owners were instructed on how to administer injections. The possibility of local reactions should always be considered, and owners should be instructed to monitor the sites of injections. Noteworthy are the severe systemic reactions characterized by a reluctance to move, tremors and generalized pain affecting mostly small breed dogs and resolving with treatment discontinuation.

One dog developed acute pancreatitis with severe symptoms, requiring treatment suspension and hospitalization. Acute pancreatitis had already been described in rare single cases [18,31]. However, in a study of 30 dogs, specific canine pancreatic lipase concentration (cPLI) did not show a significant increase during aNm treatment, and no signs or alterations attributable to pancreatitis were recorded [30].

Based on the available literature, there are conflicting data concerning the possibility that aNm may induce renal damage and worsen an already compromised renal situation. Some studies have documented a worse impact of aNm on kidneys, evaluated through biochemical parameters and urine examination, in comparison with MIL [8,17,18,20]. Tubular damage was identified through renal biopsy in healthy animals treated with aNm but not in those treated with MIL [19]. On the other site, some studies have reported no impact of aNm on renal function [6,9,12,21]. Furthermore, the use of aNm in patients with documented renal injury associated with CanL is a highly debated issue, but most of the authors suggest that in patients with advanced renal impairment, the use of aNm may be contraindicated [12,27] and in this circumstance, an alternative pharmacological treatment such as MIL is often preferred. Our retrospective study again calls for caution in the use of aNm in patients with renal damage, as three dogs presented worsening renal function parameters associated with clinical signs of renal failure. Moreover, one dog developed severe renal failure, despite normal renal function before treatment (LeishVet stage II). Daza Gonzáles et al. [12] already described the development of acute renal failure after treatment in a single dog and attributed it to antimonate toxicity inducing tubular necrosis or to glomerular disease related to immunocomplex deposition due to rapid parasite death. The dog in our study, a 2-year-old male Dogue de Bordeaux, showed sudden lethargy, anorexia, and weakness about 1 week after the beginning of aNm treatment. The dog’s renal condition never reverted to normal and the dog died 1 month later. The authors are more prone to consider aNm-associated direct tubular damage rather than damage due to the rapid death of amastigotes with the development of an acute immune-mediated glomerulopathy because of the absence of proteinuria. Acute pancreatitis and tubular renal damage are rare but possible, probably due to individual predisposition or unknown concomitant factors.

In the prospective study, we decided to select a population of dogs with clinical leishmaniasis associated with no or mild compromised renal function (LeishVet stage II and III, CKD IRIS 1). This decision was made due to ethical reasons and based on the retrospective study results, showing that the impact of aNm on subjects with compromised renal function can lead to clinical worsening. In this selected population, none of the dogs presented worsening of renal function during or after treatment. Only one dog (14) showed temporary hyperazotemia at D180, but timing excludes the hypothesis of an association with aNm.

Regarding the interpretation of UPC, proteinuria was considered of renal–glomerular origin in the absence of urinary active sediment. In most dogs with CKD IRIS 1 (UPC > 1), treatment with aNm was associated with a reduction in proteinuria as previously reported by other authors [12,21,32]. In two dogs, a temporary increase in UPC at D30 was observed (dog 3 and 16). Transient increments in proteinuria had already been reported and attributed to the release of antigens after the parasite’s death [33].

From our prospective study data, we can deduce that in dogs with normal renal function or with CKD IRIS 1, treatment with aNm had no negative impact on renal function itself. On the other hand, data from our retrospective study suggest caution in the use of aNm, particularly in subjects with advanced renal injury and/or compromised renal function and again document that acute renal damage can be a possible but rare event in single dogs with normal renal function.

Noteworthy are the severe idiosyncratic cutaneous reactions not previously reported to the author’s knowledge. It is interesting that in the three dogs presented for consultation due to the rapid onset of cutaneous signs under treatment, therapy was not immediately discontinued because neither the owners nor the referral vet quickly associated them with aNm adverse events. Treatment discontinuation allowed complete recovery in two dogs. On the contrary, in the third dog, despite treatment suspension, the process progressed. In this extremely severe case, it is possible to hypothesize that immune-mediated vasculitis probably due to the rapid death of parasites could have contributed to the dramatic evolution. Cutaneous vasculitis has already been described as a post-treatment complication in CanL [9].

Due to the severity of some reported conditions, it is critical that clinicians and owners are aware of the possibility of immediately discontinuing treatment if unexpected signs appear during therapy.

Concerning aNm clinical efficacy, the results of our prospective study confirm what has already been reported by numerous authors, including the following: clinical score improvement already evident at the end of treatment, normalization of hematobiochemical alterations, and absence of clinical relapses until the end of the study.

It was not surprising that IFAT titers after treatment slowly reduced in most of the dogs without seroconversion in any dog. The relevance of monitoring IFAT titers for disease follow-up and post-treatment monitoring has long been controversial [22], but common opinion is now that it is of limited value. In fact, IFAT does not show any significant correlation with the clinical status of the animals and in some subjects, the antibody titers may remain elevated for years after treatment, particularly in endemic areas [9,34].

The most interesting finding of our study is the high number of dogs (78.6%) that tested negative with qPCR at D60, suggesting that the parasitological clearance after treatment could be more frequent than previously suggested; in fact, it is reported in the literature as a possible but rare event under leishmanicidal treatment [1]. Efficacy of the aNm tested protocol in terms of parasitic clearance was very high in our study. In a recent study by our own research group that aimed to compare two dosing regimens of MIL [7], and working with a similar study design and dog population, bone marrow qPCR was negative in 50% and 14.3% of the dogs treated with a modified or standard dose of MIL, respectively [7]. The high percentage of parasitic clearance after treatment leads the authors to suggest that the potential side effects do not justify discarding the aNm protocol, which remains an excellent option at least in the absence of renal damage.

A limitation of the retrospective study is that the adverse effects were extrapolated based solely on the presence of clinical manifestations. Any possible subclinical laboratory alterations presented during treatment were not investigated. Only in dogs for whom clinical adverse events were registered, collateral tests were evaluated for a better understanding of the condition (i.e., renal failure, pancreatitis).

A limitation of the prospective study is the limited number of dogs and the lack of a specific control group. To address the latter limitation, the authors referred to a previous work of the same research group to compare efficacy in terms of parasitic clearance.

## 5. Conclusions

In conclusion, treatment with aNm in dogs with CanL can be associated with mild to severe adverse events including local, systemic and idiosyncratic reactions, and can have a potential impact on renal function, especially if already compromised. Veterinarians must be vigilant regarding the potentially serious adverse effects associated with aNm and immediately stop drug administration if unexpected clinical manifestations occur. Nevertheless, it is the authors’ opinion that the aNm protocol should still be proposed as the first-choice treatment for CanL, at least in dogs with preserved renal function and in CKD IRIS 1. Monitoring during treatment and owner compliance are essential to its safe use; what does not kill you makes you stronger!

## Figures and Tables

**Table 1:** Signalment data of the 87 dogs included in the retrospective study.

		Breed	
		Mongrel	26 (29.9%)
**Females**	n: 40 (46%)	English setter	12 (13.8%)
**Males**	n: 47 (54%)	German Shepherd	7 (8%)
		Doberman pinscher	7 (8%)
		Great Dane	4 (4.6%)
		Beagle	4 (4.6%)
		Boxer	4 (4.6%)
**Age (years)**	Mean: 5.25 (Min: 0.75; Max: 14)	Breton Spaniel	3 (3.4%)
**Weight (kg)**	Mean: 22.47 (Min: 4.5 Max: 50)	English pointer	2 (2.3%)
		Labrador retriever	2 (2.3%)
		Pomeranian	2 (2.3%)
		Others *	14 (16%)

* One dog for each of the following breeds: American Pit Bull Terrier, Border Collie, Cocker Spaniel, Argentine mastiff, Golden Retriever, Greyhound, Husky, Italian hound, Italian Spinone, Little Italian Greyhound, Maremma Sheepdog, Pinscher, Shar Pey; Weimaraner.

**Table 2:** Clinical signs and/or presenting complaints at diagnosis in the 87 dogs included in the retrospective study.

Clinical Signs	Dogs n	% (tot 87)
Lymphadenomegaly	69	79.3
Dermatological signs	61	70
Weight loss	48	55.2
Pale mucous membranes	14	16
Ophthalmological signs	13	15
Lameness	9	10.3
Dysorexia	7	8.1
Splenomegaly	7	8.1
Lethargy	6	6.9
Epistaxis	5	5.7
Ophthalmological signs	5	5.7
Gastrointestinal signs	5	5.7
Joint swelling	5	5.7
Abdominal pain	4	4.6
Others *	31	35.6

* “Other” clinical signs/presenting complaint at the time of diagnosis include unilateral epistaxis (n = 3), lameness (n = 3), polyuria/polydipsia (n = 3), respiratory sounds (n = 3), unformed soft stools (n = 2), polyuria/polydipsia (n = 2), tonsillitis (n = 2), tremors (n = 2), gingivitis (n = 1), and sneezing (n = 2), oral cavity mouth ulcer (n = 1), posterior train pain (n = 1), vulvar discharge (n = 1), frontal muscle atrophy (n = 1), cough (n = 1), hyperthermia (n = 1), muscular hypotrophy (n = 1), and dysorexia (n = 1).

**Table 3:** Number and relative percentage of dogs that developed clinical signs and pathological conditions during treatment associated with N-methylglucamine antimoniate (aNm) adverse events; number and relative percentage of dogs in which treatment was discontinued.

Clinical Signs/Pathological Conditions	Dogs n (tot 87)	% (tot 87)	Treatment Suspension (Dogs n)	% (tot 87)
Local reaction at the site of inoculation	6	6.9	1	
Diarrhea	5	5.7	1	
Severe systemic reaction to pain originating from the inoculation site associated with lethargy, anorexia, and reluctance to move	4	4.6	3	
Severe systemic reaction due to renal failure	4	4.6	4	
Vomiting	3	3.4	0	
Severe cutaneous idiosyncratic reactions	3	3.4	3	
Severe systemic reaction due to acute pancreatitis	1	1.1	1	
**Total**	26	29.8	13	14.9

**Table 4:** Dogs’ data, clinical score, laboratory parameters, and LeishVet classification at the time of inclusion in the prospective study. Dog 5 and dog 12 were excluded from the study before D30 due to severe local reaction.

Dog	Breed	Sex/Age (Years)/ Weight (kg)	Clinical Score	HCT (%)	RBC (×1000/µL)	HGB (gr/dL)	TP (gr/dL)	Alb (gr/dL)	Glob γ (%)	Glob γ (gr/dL)	A/G	IFAT Titer	Urea (mg/dL)	Crea (mg/dL)	UPC	LeishVet Clinical Stage
C1	Mongrel	M/8/32	5	42	7.34	14.5	9	2.07	38.3	3.45	0.3	320	28	0.99	0.26	II
C2	Mongrel	F/3/20	14	40.3	6.57	13.5	13	2.31	61.8	8.03	0.22	320	31	1.02	0.61	II
C3	Mongrel	M/9/30	9	42.4	6.87	14.3	9	2.67	33.5	3.02	0.42	320	34	1.06	0.28	II
C4	German Sheperd	F/6/18	11	38.3	5.97	13.2	7.5	2.66	27.2	2.04	0.55	320	58	1.01	1.1	II
C5	Mongrel	M/7/13	6	40.6	6.14	13.4	11.3	2.85	45.7	5.16	0.34	320	40	1.29	1.1	III IRIS 1
C6	Boxer	F/4/26	10	28.2	4.49	9.2	10.2	1.91	55.4	5.65	0.23	320	30	0.78	3.55	III IRIS 1
C7	Rottweiler	M/6/20	13	30.8	4.65	10.1	8.4	2.27	39.6	3.33	0.37	320	21	0.83	0.4	II
C8	Setter	M/5/17	6	44.6	6.96	15.2	7.5	2.81	36.3	2.72	0.6	320	39	1.07	1.59	III IRIS 1
C9	Mongrel	M/8/13	5	43.1	6.45	14.6	8.1	3.02	14.6	1.18	0.59	320	55	0.95	0.4	II
C10	German Sheperd	F/7/25	7	38.5	6.08	12.8	7.3	2.47	21.6	1.58	0.51	320	32	1.13	0.18	II
C11	Italian Spinone	M/5/20	9	32.9	4.93	10.6	9.2	2.60	38.8	3.57	0.39	320	46	1.07	1.62	III IRIS 1
C12	Labrador	M/12/30	10	24.8	3.80	7.2	10	18.2	53.5	5.32	0.22	320	41	0.78	0.96	III IRIS 1
C13	Mongrel	M/8/22	15	33.9	5.38	11.1	9.4	2.48	46.2	4.34	0.36	320	30	0.95	0.94	II
C14	Mongrel	F/7/18	11	32.5	5.20	10	9	1.75	49.1	4.42	0.24	320	32	1.18	1.49	III IRIS 1
C15	Mongrel	F/5/4.8	12	28.2	4.45	9.4	9.2	2.94	34.3	3.16	0.47	320	28	0.87	1.24	III IRIS 1
C16	Mongrel	F/8/12	22	27.3	4.28	8.3	8.2	1.41	42.3	3.47	0.21	320	24	0.6	1.1	III IRIS 1

**Table 5:** Urine protein/creatinine ratio (UPC), urea, and creatinine parameters of the 16 dogs enrolled in the prospective study evaluated at D0, D30, and D60 to assess the renal impact of N-methylglucamine antimoniate (aNm) treatment. Dogs 5 and 12 were excluded from the study before D30 due to severe local reaction.

	UPC			UREA			CREATININE		
DOGS	D0	D30	D60	D0	D30	D60	D0	D30	D60
**C1**	0.26	0.24	0.33	28	31	32	0.99	1.04	1.08
**C2**	0.61	0.27	0.14	31	43	26	1.02	0.89	0.27
**C3**	0.28	0.97	0.23	34	16	28	1.06	0.69	0.98
**C4**	0.21	0.17	0.17	58	32	27	1.01	0.71	0.89
**C5**	1.1			40			1.29		
**C6**	3.55	1.66	0.39	30	21	15	0.78	0.85	0.95
**C7**	0.4	0.61	0.28	21	19	23	0.83	0.84	0.8
**C8**	1.59	0.24	0.24	39	24	34	1.07	0.96	1.08
**C9**	0.4	0.47	0.55	55	28	30	0.95	0.77	0.73
**C10**	0.18	0.23	0.27	32	28	36	1.13	1.04	1.02
**C11**	1.62	0.51	0.2	46	38	38	1.07	0.9	0.89
**C12**	0.96			41			0.78		
**C13**	0.94	0.33	0.47	30	32	25	0.95	0.99	1.13
**C14**	1.49	0.31	0.5	32	22	43	1.18	1.01	1.31
**C15**	1.24	0.89	0.95	28	23	45	0.87	0.76	0.9
**C16**	1.1	2.98	0.83	24	40	38	0.6	0.88	0.86

**Table 6:** **q**PCR results on bone marrow at D0 and D60 of the 14 dogs included in the prospective study. In dogs 5 and 12, PCR was not performed since both patients were excluded from the study.

DOG	D0	D60
C1	1.1 × 10^3^	(−)
C2	3.3 × 10^5^	(−)
C3	1.3 × 10^4^	(−)
C4	1.1 × 10^3^	(−)
C6	8.7 × 10^4^	(−)
C7	(−)	(−)
C8	3.5 × 10^4^	1.7 × 10^4^
C9	2.8 × 10^5^	(−)
C10	2.3 × 10^5^	2.4 × 10^2^
C11	1.1 × 10^6^	(−)
C13	9.2 × 10^5^	(−)
C14	6.6 × 10^6^	(−)
C15	6.7 × 10^5^	(−)
C16	6.0 × 10^6^	1.7 × 10^4^

## Data Availability

The dataset is available from the authors.
